# Mild Clinical Presentation of Acute Fatty Liver in the Second Trimester of Pregnancy

**DOI:** 10.1155/2011/402710

**Published:** 2011-08-18

**Authors:** Alaeddine Yassin, Walid Denguezli, Anissa Fessi, Leila Njim, Raja Falah, Abdelfattah Zakhama, Mohamed Sakouhi

**Affiliations:** ^1^Department of Obstetric and Gynecology, Fattouma Bourguiba Teaching Hospital, Avenue June 1, Monastir 5000, Tunisia; ^2^Department of Anatomy and Cytopathology, Fattouma Bourguiba Teaching Hospital, Avenue June 1, Monastir 5000, Tunisia

## Abstract

We report a case of 29 years old woman who was diagnosed with acute fatty liver of pregnancy at 23 weeks of gestation with unusual evolution (pregnancy prolonged until 36 weeks of gestation) to draw attention on the possibility of occurrence of this pathology in the second trimester of pregnancy even with a milder clinical presentation and course.

## 1. Introduction

Acute fatty liver of pregnancy (AFLP) is an uncommon, life-threatening complication that occurs in the third trimester or early postpartum period [1]. In the literature, only few cases of acute fatty liver of pregnancy in the second trimester were reported [2, 3]. Here, we report a case of AFLP with a milder clinical presentation in the second trimester.

## 2. Case Report

**Figure 1:**
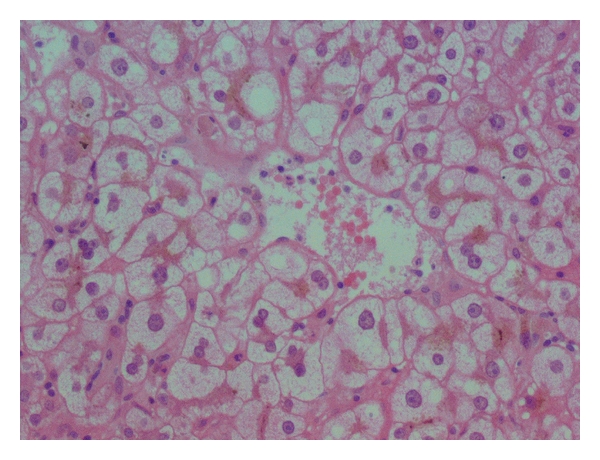


A 29-year-old woman, gravid 1, presented at 23 weeks' gestation with nausea, vomiting, abdominal pain, malaise, and weight loss. An ultrasound at 11 weeks' gestation determined gestational age. She was normotensive with a normal platelet count and no proteinuria. She had elevated liver transaminase levels (aspartate aminotransferase [ASAT] level 108 U/L, alanine aminotransferase [ALAT] level 104 U/L), prothrombin time [PT] 96%, negative hepatitis serology (A, B, C, and E), and a negative immunologic analysis. She was treated with intravenous fluids, but we remarked a slightly aggravation of her laboratory findings ASAT 122 U/L, ALAT 248 U/L, GGT 18 U/L, PAL 118 U/L, bilirubin total/direct 23,2/17,4 *μ*mol/L, PT 76%, WBC 7800, platelets 178000, glucose 65 mg/dL, and uric acid 246 *μ*mol/L. The abdominal ultrasound showed a brilliant liver without others anomalies. Because of the doubt on diagnosis and stable clinical and biologic parameters, a liver biopsy was performed (10 days after admission) and showed a microvesicular fatty infiltration of the hepatocytes (Figure 1). A diagnosis of acute fatty liver of pregnancy was confirmed. The decision was to prolong the pregnancy under surveillance in obstetric unit care with clinical and biological control. The biological analyses were stable (Table 1) with good clinical condition. The evolution was good and the pregnancy was prolonged until 36 weeks of gestation, and the patient gave birth to a healthy newborn of 2900 g, Apgar score of 9/10. The delivery was simple without any complications. After delivery, all biologic parameters were normalized within 7–10 days and the patient was discharged.

## 3. Comments

Acute fatty liver of pregnancy is a rare and potentially fatal condition. It has been thought that this condition is rare and usually takes a devastating course with significant morbidity and mortality [1], incidence is estimated to be 1/1000 to 1/20000 deliveries [1], and, generally, it occurs in the third trimester or early postpartum period of pregnancy, but a few cases in the second trimester were published [2, 3]. Furthermore, modern advances in diagnostic capabilities have given us a better understanding of the pathological process, and many cases with a milder clinical course as well as subclinical cases have been reported [4–6]. Our observation is unusual by its clinical evolution until term and time of arising (second trimester). Indeed, Monga and katz [2] reported a case of AFLP arising at 22 weeks' gestation in 35 years women. However, in this case evolution was interrupted because of a disseminated intravascular coagulation.

Also, Suzuki et al. [3] reported a case arising at 23 weeks of gestation for which the interval between symptom occurrence and fetal demise was only one week and pregnancy was quickly interrupted.

Curiously, biological findings in our observation were constant during evolution of pregnancy, particularly, prothrombin test and platelet counts. These findings may confirm the cases with subclinical or milder clinical course already reported.

Usually, there is no specific treatment for acute fatty liver of pregnancy except prompt termination of pregnancy and pathological changes as a rule improve rapidly after delivery. Liver biopsy is the best way to confirm the diagnosis, but because it is invasive, it is not always performed. Furthermore, today we can take advantage of noninvasive procedures to demonstrate fat in the liver and to exclude other liver diseases such as viral hepatitis [7]. Histopathological finding in liver biopsies is small and midsized intracellular vacuoles, without displacement of the nucleus and ballooning hepatocytes.

Recently, an association between AFLP and a deficiency of the enzyme long-chain 3-hydroxyacyl-CoA dehydrogenase was suggested. This enzyme is one of the four enzymes, which break down long-chain fatty acids in the liver. Deficiency of this enzyme results in the increased accumulation of long-chain fatty acids [8]. However, the exact mechanism that explains these mild clinical presentations remains unknown.

This case, one of few reported cases, shows that even in the second trimester, the diagnosis of acute fatty liver should be considered when a woman presents with deteriorating liver function, jaundice, and coagulopathy. The particularity of this case is the unusual evolution; with a stable clinical and biological perturbations for 13 weeks after diagnosis of AFLP. Finally, the diagnosis of AFLP must be suspected in front of all digestive symptomatology in the second or the third trimester of pregnancy.

## Figures and Tables

**Table 1:** Biologic analysis evolution.

Day after admission	1	3	7	21	42	63	84	91
ALAT IU/L	104	248	213	165	172	146	134	145
ASAT IU/L	108	122	104	106	110	96	98	102
PAL IU/L	138	118	123	121	142	134	129	119
BIL T *μ*mol/L	14	23,2	18,4	16,7	14,5	16	15,8	17,3
BIL D *μ*mol/L	8,5	17,4	12,6	11,2	10,4	12,1	11,3	12,9
Creatinine *μ*mol/L	64	78	67	56	64	61	73	57
Uric acid mmol/L	144	138	145	164	154	151	167	173
Hemoglobin g/dL	12,2	12,3	11,9	12	11,5	11,7	11,4	11,8
Platelets/mm^3^	213000	178000	198000	187000	201000	221000	197000	184000
WBC	7600	7800	8500	9200	7400	10100	10700	12000
PT %	96%	76%	78%	81%	92%	84%	79%	85%
Glucose mmol/L	3,6	4	4,9	5,1	4,4	5,3	4,8	5,5

WBC: white blood cell.

PT: prothrombin time.

ASAT: aspartate aminotransferase.

ALAT: alanine aminotransferase.
